# Cancer-associated fibroblast infiltration in osteosarcoma: the discrepancy in subtypes pathways and immunosuppression

**DOI:** 10.3389/fphar.2023.1136960

**Published:** 2023-06-27

**Authors:** Zhang Zhihao, Ju Cheng, Zuo Xiaoshuang, Ma Yangguang, Wu Tingyu, Yang Yongyong, Yao Zhou, Zhou Jie, Zhang Tao, Hu Xueyu, Wang Zhe

**Affiliations:** ^1^ Department of Orthopedics, Xijing Hospital, Air Force Military Medical University, Xi’an, Shaanxi, China; ^2^ Department of Radiation Oncology, National Cancer Center, National Clinical Research Center for Cancer, Cancer Hospital, Chinese Academy of Medical Science and Peking Union Medical College, Beijing, China

**Keywords:** osteosarcoma, cancer-associated fibroblasts, cancer genome atlas, immune infiltration, drug resistance

## Abstract

**Introduction:** Osteosarcoma (OS), the primary malignant bone tumor, has a low survival rate for recurrent patients. Latest reports indicated that cancer-associated fibroblasts (CAFs) were the main component of tumor microenvironment, and would generate a variable role in the progression of tumors. However, the role of CAFs is still few known in osteosarcoma.

**Methods:** The processed RNA-seq data and the corresponding clinical and molecular information were retrieved from the Cancer Genome Atlas Program (TCGA) database and processed data of tumor tissue was obtained from Gene Expression Omnibus (GEO) database. Xcell method was used in data processing, and Gene set variation analysis (GSVA) was used to calculates enrichment scores. Nomogram was constructed to evaluate prognostic power of the predictive model. And the construction of risk scores and assessment of prognostic predictive were based on the LASSO model.

**Results:** This study classified Cancer Genome Atlas (TCGA) cohort into high and low CAFs infiltrate phenotype with different CAFs infiltration enrichment scores. Then TOP 9 genes were screened as prognostic signatures among 2,488 differentially expressed genes between the two groups. Key prognostic molecules were CGREF1, CORT and RHBDL2 and the risk score formula is: Risk-score = CGREF1*0.004 + CORT*0.004 + RHBDL2*0.002. The signatures were validated to be independent prognostic factors to predict tumor prognosis with single-factor COX and multi-factor COX regression analyses and Norton chart. The risk score expression of risk score model genes could predict the drug resistance, and significant differences could be found between the high and low scoring groups for 17-AAG, AZD6244, PD-0325901 and Sorafenib.

**Discussion:** To sum up, this article validated the prediction role of CAF infiltration in the prognosis of OS, which might shed light on the treatment of OS.

## 1 Introduction

OS is one of the most prevalent primary malignant bone tumors, which occurs principally in kids, teenagers and young adults (Keil, 2020). Operative resection and combined chemotherapy treatment can cure about 70% of patients, and the 5-year survival rate of patients with limited osteosarcoma has increased significantly in the past decades (Kansara et al, 2014; Robison and Hudson, 2014). However, as a highly aggressive tumor, the 5-year survival rate for patients with recurrent and metastatic OS remains at approximately 20%, virtually constant for the past 30 years (Kansara et al, 2014). Therefore, the treatment of osteosarcoma still requires the application of new therapies.

It has been shown that the tumor microenvironment is actively involved in tumor progression (Liotta and Kohn, 2001; Mueller and Fusenig, 2004). The activated mesenchymal cells, CAFs, are a major component of the tumor microenvironment (Chen and Song, 2019). Compared to normal fibroblasts, multiple protein markers are overexpressed in CAFs depending on the tumor type such as α-smooth muscle actin (α-SMA) or fibroblast activation protein (FAP) (Kalluri and Zeisberg, 2006). CAFs could interact with tumor cells by releasing secreted proteins such as transforming growth factor β (TGF-β), insulin-like growth factor (IGF) and interleukin-6 (IL-6), regulating immune feedback or remodeling of the extracellular matrix, etc. (Ishii et al, 2016). Meanwhile, it was demonstrated that the composition of CAFs is heterogeneous, different degrees of CAFs activation would generate different subgroups of CAFs and play a variable role in the progression of tumors (Wang et al, 2021).

OS is a low immunogenic tumor that is less likely to induce an immune response in the host by contrast to immunotherapy effective cancers such as malignant melanoma and lung cancer (Yahiro and Matsumoto, 2021). Immunogenicity is determined by what is known as the tumor mutation burden (TMB), i.e., the accumulation of mutations in the tumor. As a type of sarcoma, OS has a low TMB value. Immunogenicity will largely determine the effectiveness of immunotherapy, and low immunogenicity of OS would result in fewer immune cells tumor-infiltrating and tumor-specific T-cells, making immunotherapy ineffective (Wang et al, 2019a; Yahiro and Matsumoto, 2021). Over the past few decades, the tumor microenvironment has been recognized as a rich target for anti-tumor therapy (Turley et al, 2015), Numerous preclinical studies have also shown that CAFs could be a potential promising target for anti-tumor immunotherapy (Kalluri, 2016; Ziani et al, 2018; Kobayashi et al, 2019).

In osteosarcoma, the studies on the role of CAFs are still few in quantity. Here, as detailed flow chart of this study demonstrated, data derived from TCGA and GEO were divided into high and low fibroblast groups according to CAFs infiltration enrichment scores, and patients in the high fibroblast group had significantly longer OS survival time than those in the low fibroblast group, they differed significantly in stromal and immune-related scores. Then, differentially expressed genes (DEGs) were further screened and enriched for pathways in the GO database of MF, BP, CC, and KEGG, and most of the differentially expressed genes were found to be enriched for immune-related pathways. Next, based on univariate COX regression analysis, 9 genes significantly associated with overall patient survival were screened and identified as fibroblast-related risk markers, and three of them were screened as prognostic molecules. Assigning prognostic molecules with different weights, the risk score of each sample was calculated and samples were divided into high risk and low risk groups based on the median, and the prognostic difference between them was verified using ROC curves. The risk score could be used as an independent predictor of prognosis. For patients with higher risk scores, there was a tendency for higher tumor microenvironment scores and significantly altered tumor hallmark pathway genes. Finally, comparing the IC50 of the drugs, there was a significant difference in the drug sensitive profile between the high and low scoring groups.

## 2 Materials and methods

### 2.1 Dataset and source

**FIGURE 1:**
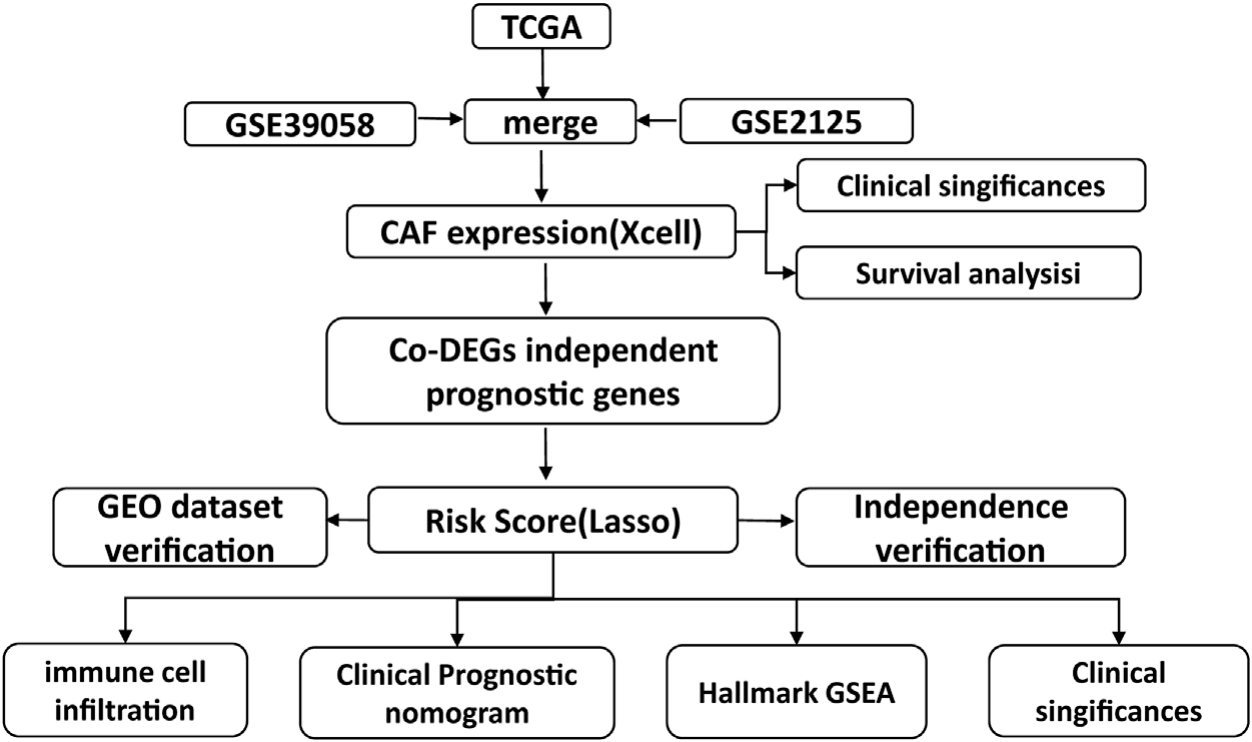
Flow chart of the experiment.

In this study, we used TCGA (https://portal.gdc.cancer.gov/) and GEO (https://www.ncbi.nlm.nih.gov/geo) (GSE21257 and GSE39058) platforms for data analysis, and the data of the two platforms were used respectively. First, the processed RNA-seq data (88 samples, 85 survival data) and the corresponding clinical and molecular information were retrieved from the TCGA database. After excluding 90% of the NA fields in clinical information, race, gender, age, and site_of_resection_or_biopsy were selected as clinical characters, 85 patients from TCGA cohort were included. The analysis was performed by TCGA data for subtype exploration, prognosis-related gene screening and prognostic model construction. Then we validated the prognostic model using GEO microarray data. Processed data of tumor tissue from patients with osteosarcoma was downloaded from NCBI GEO with accession code GSE39058 and GSE2125. Age, gender, and recurrence were selected as clinical characters in GSE39058 cohort and metastases, and huvos.grade for GSE2125 cohort. Data from the two cohorts of the GEO platform were merged by the COMBAT function in the R sva package. The same type of clinical information (age, gemder) was combined in a clinical correlation analysis. Each of the GSE39058 and GSE2125 cohorts corresponded probes to genes based on information from their corresponding microarrays, and empty vector probes were removed. If multiple probes corresponded to one gene, we selected the median of these probes as the expression level of that gene. As a result, 95 patients from GSE39058 and GSE2125 cohort were included in our study. The flow chart of the experiment could be found in Figure 1.

### 2.2 Data preprocessing

The downloaded TCGA expression profile data in FRKM format was converted to TPM format. The EPIC, MCP-counter, and Xcell methods were used to calculate the percentage of immune cell infiltration in each sample. The results of the K-M analysis and the one-way COX analysis were compared between above three methods and the Xcell method was selected as it had the best results. To analyze the differences between patients with high CAF scores and low CAF scores, we used the median of the CAF score-based cohort to divide the patients into the high CAF and low CAF groups to reduce the bias caused by the different numbers in the two groups.

### 2.3 GSVA pathway analysis

GSVA (Gene set variation analysis) is a non-parametric, unsupervised algorithm that calculates enrichment scores for specific gene sets in each sample without pre-grouping the samples. By using the MSigDB-hallmark gene as the reference gene set and set *p*-value to <0.05, we performed GSVA and implemented using package clusterProfiler in R. The commonly activated or suppressed pathways were identified.

### 2.4 Nomogram construction

Clinical information and risk scores including age, gender and tissue origin included in the TCGA cohort were selected for nomogram development, the prognostic risk score models were built using the RMS package. Performance of the model was validated in the TCGA cohort using time-dependent calibration curves and Harrell’s concordance index (C-index) to assess the prognosis validity of the model.

### 2.5 Identification of prognosis-related genes

COX regression analysis and K-M survival analysis were performed to identify the genes which might related to overall survival in database and evaluated the contribution of the genes, *p <* 0.05 was considered as statistically significant. The analysis was conducted with the package of survival and survminer.

### 2.6 Constructing risk scores and prognostic predictive assessments based on the lasso model

Interactions between genes might form covariate gene clusters, we use R/Bioconductor’s lars and glmnet R language package for Lasso regression to reduce the impact of this covariation and improve the accuracy and interpretability of the model. Then we used cross-validation to determine the corresponding parameters in order to obtain a suitable model within the existing models. Based on the obtained model, we calculated the risk score (Risk score = $\sum_{i=1}^{N}coef_{i}*\exp r_{i}$ ) for each patient, in which N is the number of genes selected, expr_i_ is the expression value of each gene and coef_i_ is the multivariate COX regression coefficient. The cohort was divided into low-risk and high-risk groups based on the median risk score, and survival analysis was performed between the two groups using the Kaplan-Meier progression. ROC curves were plotted to predict prognostic 1-, 3-, and 5-year survival rates for patients with osteosarcoma using the survivalroc R package.

### 2.7 Differential gene analysis and pathway annotation

Data from the TCGA cohort were formatted using the read count package and differential analysis was performed using the deseq2 R package to screen for differential genes between high and low risk scores (*p*-value <0.01 and abs (log_2_FoldChange) > 1). Differential analysis was performed on the GEO microarray data via the limma package and screened the differential genes as described above. Gene set enrichment analysis (GSEA) was performed using the clusterProfiler R language package, with the using of “h.all.v7.0.entrez.gmt” as the reference gene set. *p*-values were adjusted using the Benjamini and Hochberg methods, and *p*-values <0.05 were considered as statistic significant.

### 2.8 Drug resistance assessment by CAFs score

Firstly, based on the expression profile matrix of 471 cell lines downloaded from Cancer Cell Line Encyclopedia (CCLE), the CAFs scores in each cell line sample were calculated by CAFs score model, then the cohort in each sample was divided into high-CAFs group and low-CAFs group based on median, then the resistance IC50 assessment of 471 samples with 24 drugs was corelated, and whether there was a significant difference in drug resistance between the two groups was tested by Wilcoxon signed rank test.

### 2.9 Cell culture and CCK-8 test

HOS and MG-63 cells were purchased from Meisen Cell Technology Co., Ltd. Cells were cultured in Dulbecco’s modified Eagle medium (DMEM) (Gibco, United States) containing 10% fetal bovine serum and 100 units/mL penicillin-streptomycin. The viability of cells after treatment with 17-AAG (MCE, United States), AZD6244 (MCE, United States), PD-0325901 (MCE, United States), Sorafenib (MCE, United States) was determined using Cell Counting Kit-8 (CCK-8) kit (Beyotime, China). HOS and MG-63 cells were inoculated in 96-well cell culture plates at a density of 5×10^4^ cells/mL and incubated for 24 h to make cells adhere to the wall. Cells were then treated with 17-AAG, AZD6244, PD-0325901, and Sorafenib at a concentration of 1 μM for 72 h, respectively. Then 10% CCK-8 solution was added, and incubated for 2 h at 37°C. Cell viability after different drug interventions was determined by measuring absorbance values measured at 450 nm on an automated detector (BioTek, United States). All cells were incubated in an incubator at 37°C with 5% CO_2_.

### 2.10 Immunohistochemistry (IHC)

To further validate the expression of the three predicted genes, six pairs of paraffin-embedded osteosarcoma tissues and adjacent tissues were collected for IHC analysis. The study was approved by the Institutional Review Board of the First Affiliated Hospital of the Air Force Military Medical University, and all patients signed an informed consent form. All tissue sections were dewaxed, antigen-repaired, blocked, incubated with primary antibodies and secondary antibodies, and antibodies used included CGREF1 (ABclonal, A14844), RHBDL2 (GeneTex, GTX46323) and CORT (santa, sc-393108). Finally, the sections were stained using the DAB kit (CWBIO, CW 2035S) and hematoxylin. Protein expression of the three molecularly stained sections in each sample was observed by microscopy, and the positive rate relative to paracancer tissue for each IHC stained section was calculated using ImageJ software.

### 2.11 Statistical analysis

The version of the R used in this paper was R version 4.1.0 (2021-05-18)—“Camp Pontanezen”. The Wilcox method was used to analyze the scoring tests between the two groups and kruskal test was used to test for three or more groups. In the statistical plots, difference of *p* < 0.05 was considered as significant.

## 3 Results

### 3.1 Identification of CAFs infiltrate phenotype and analysis of clinical features

**FIGURE 2:**
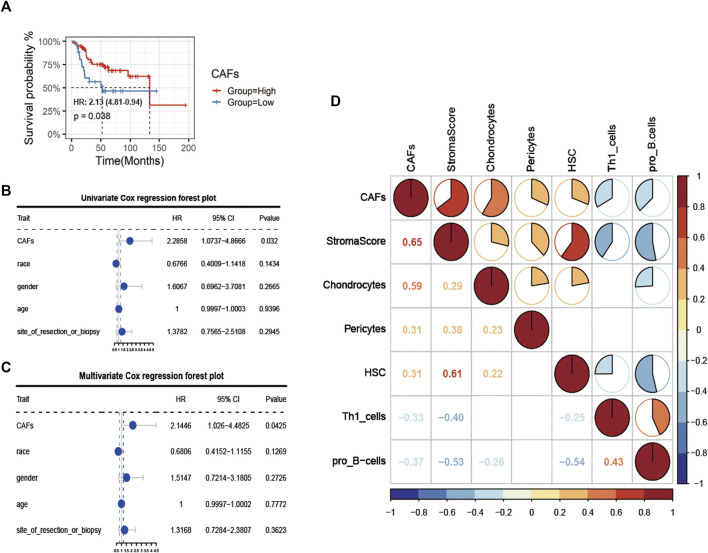
Identification of CAFs infiltration phenotype and analysis of clinical features. **(A)** K-M survival analysis between high and low CAFs score patient groups. **(B, C)** Univariate COX regression and multivariate COX regression analysis of factors including CAFs. **(D)** Correlation analysis of CAFs with immune indexes and immune-related cells.

Firstly, after obtaining CAFs infiltration enrichment scores from the TCGA cohort and applying the cut-off grouping as the median, K-M survival analysis showed a significant difference in prognostic survival between the two groups, with the high group having a better prognosis [HR (hazard rate): 2.13 (4.81-0.94)] (Figure 2A). Using univariate and multivariate Cox regression analysis with clinical information, the CAFs were shown to be independent prognostic factors (*p* < 0.05, Figures 2B,C). We further analyzed the correlation between CAFs enrichment score and other immune cell infiltration and immune correlation scores (Figure 2D), which showed a significant positive correlation between CAFs and Stroma Score, a significant positive correlation between CAFs and chondrocytes in osteosarcoma tissue, and a significant negative correlation with Th1_cells and pro_B-cells.

### 3.2 Correlation between the differences in clinical characteristics of CAFs and ESTIMATE algorithm scores

**FIGURE 3:**
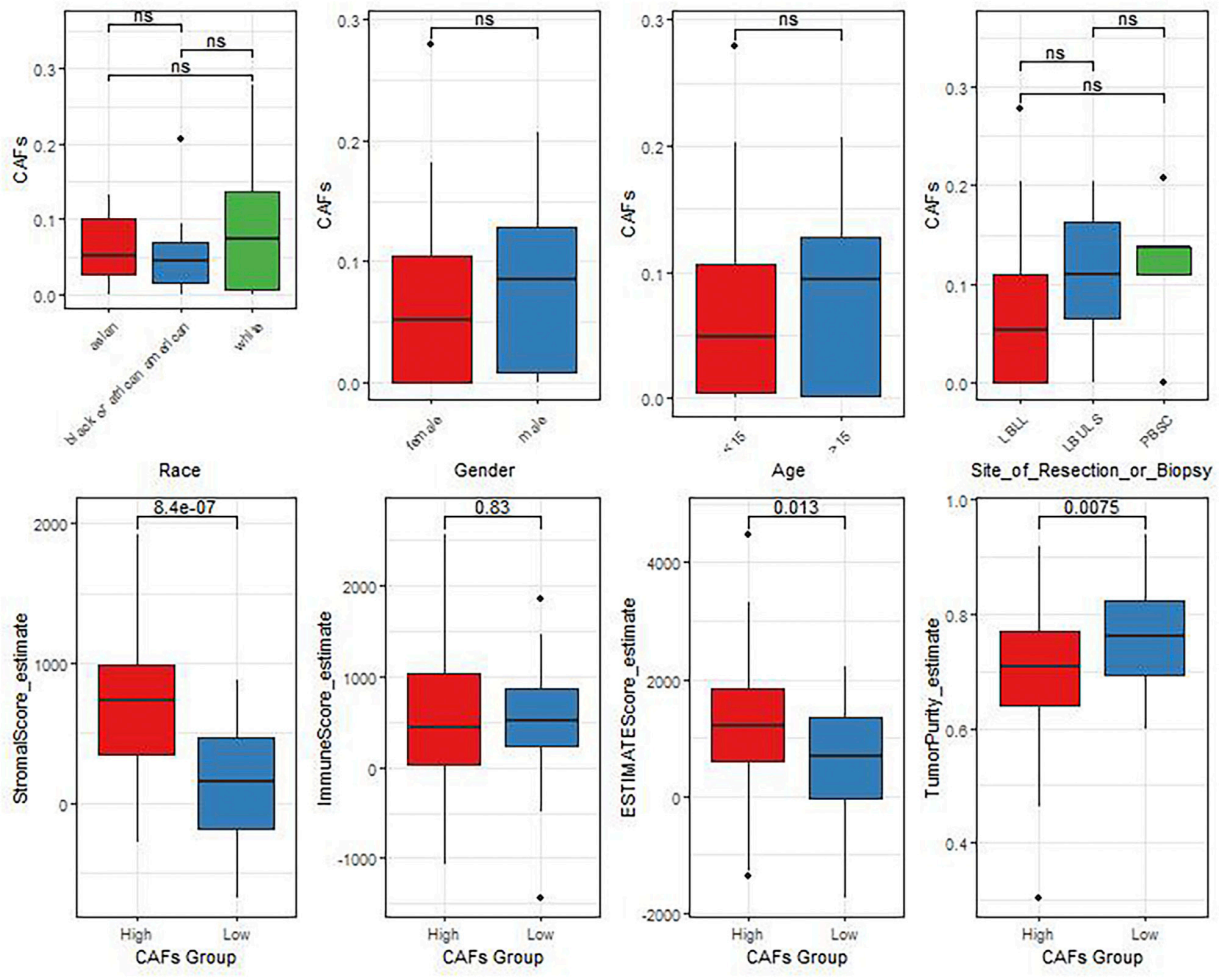
The differences of CAFs among patients with different clinical characteristics evaluated by the ESTIMATE algorithm.

In view of the crucial roles of the CAFs in tumor progression, we further analyzed the correlation between the differences in CAFs between clinical characteristics and ESTIMATE algorithm scores (Figure 3). The results showed that there were significant differences in StromalScore and ESTIMATEScore between the groups with high and low CAFs infiltrate phenotype. We also found that the low CAFs infiltration group has a higher tumor cell purity.

### 3.3 Screening of CAF candidate markers and functional analysis

**FIGURE 4:**
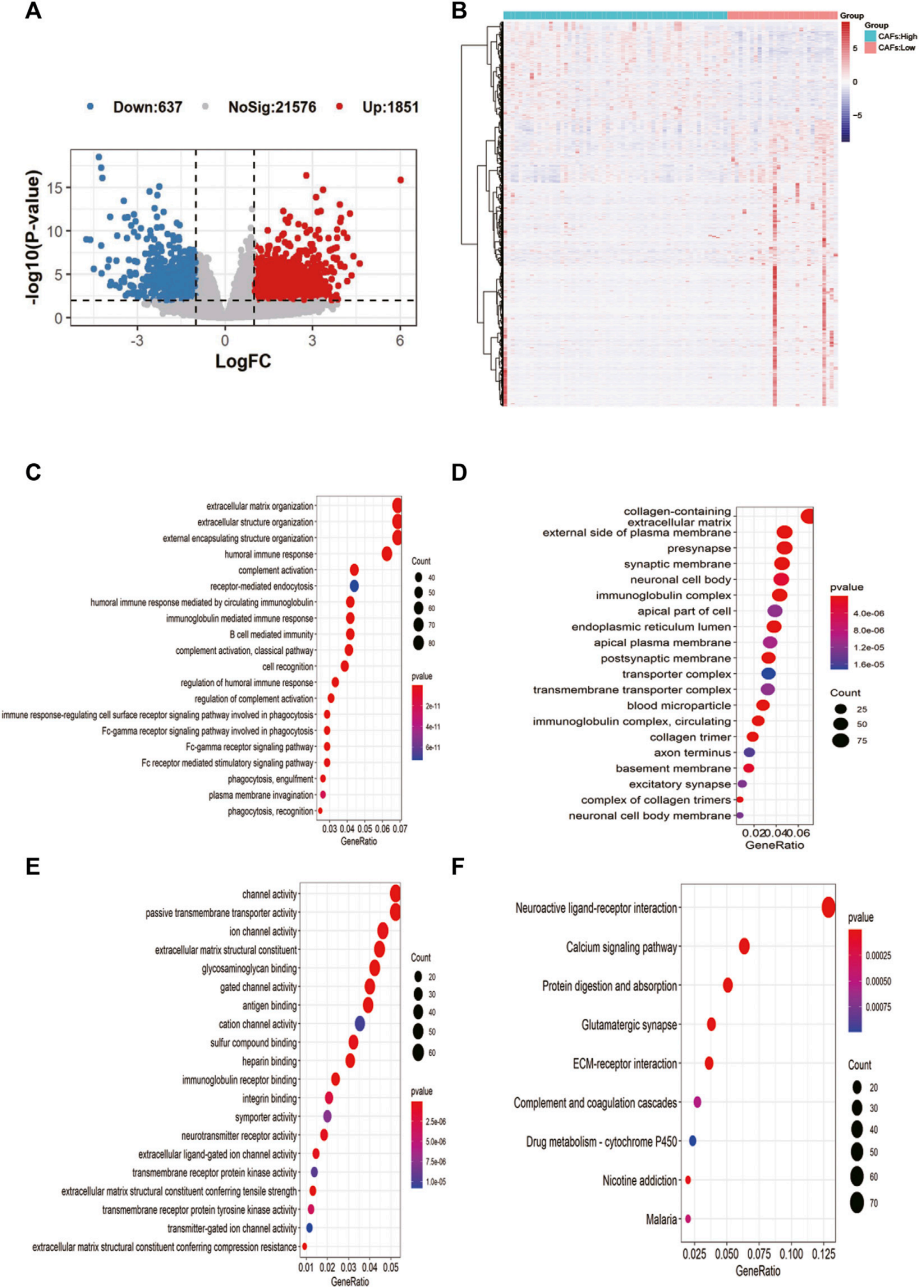
Screening of candidate gene markers of CAFs and functional analysis. **(A)** Volcano plot of differentially expressed genes between high and low CFAs score groups. **(B)** Expression profiles of differentially expressed genes between high and low CFAs score groups. **(C–E)**: Enrichment results for BP, MF, and CC pathways in the GO database. **(F)** Results of pathway enrichment in KEGG.

2,488 differentially expressed genes between the two groups were identified in TCGA, with 637 significantly upregulated and 1851 downregulated (Figure 4A). The expression heat map of the differential genes was shown in Figure 4B. The enrichment results of MF, BP, CC in the GO and KEGG showed that most of the differential genes were enriched in immune-related pathways: B cell mediated immunity, humoral immune response, etc. (Figures 4C–F).

### 3.4 Construction and validation of prognostic signatures of CAFs

**FIGURE 5:**
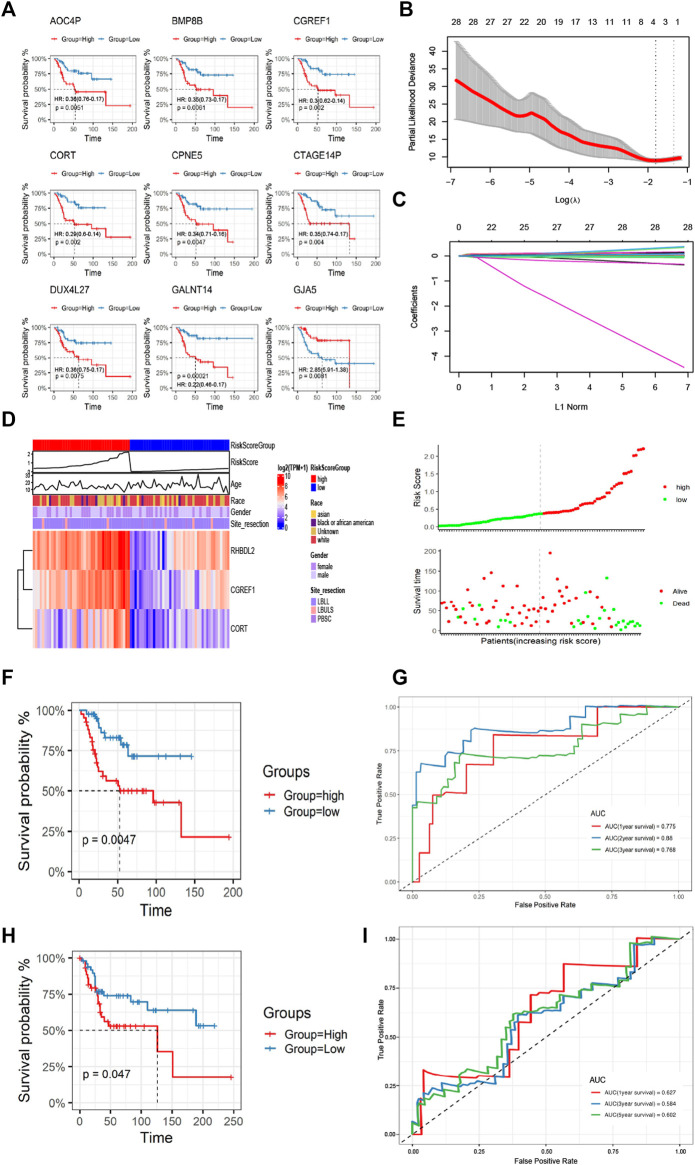
Construction and validation of CAFs prognostic signatures. **(A)** K-M survival analysis of Top 9 prognostic signatures. **(B)** A LASSO regression model was constructed using 28 candidate genes. **(C)** LASSO regression parameters were adjusted by cross validation. **(D, E)** Differences in the distribution of risk scores and prognostic signatures expression between high-risk and low-risk score groups. **(F, G)** The CAFs prognostic signatures were verified by K-M survival analysis and ROC curve drawing using TCGA training set. **(H, I)** The GSE21257 and GSE39058 cohorts were used for K-M survival analysis and ROC curve to verify the CAFs prognostic signatures.

Based on the differential gene expression profiles screened above, we used one-way COX regression analysis to screen prognostic signatures associated with patient prognosis, and TOP 9 genes including AOC4P, BMP8B, CGREF1, CORT, CPNE5, CTAGE14P, DUX4L27, GANT14, and GJA5 were selected (Figure 5A). The results of K-M survival analysis demonstrated the prognostic differences of genes [AOC4P, HR: 0.36 (0.76-0.17); BMP8B, HR: 0.35 (0.73-0.17); CGREF1, HR:0.3 (0.62-0.14); CORT, HR: 0.29 (0.6-0.14); CPNE5, HR: 0.34 (0.71-0.16); CTAGE14P, HR: 0.35 (0.74-0.17); DUX4L27, HR: 0.36 (0.75-0.17); GANT14, HR: 0.22 (0.46-0.1); and GJA5, HR: 2.85 (5.91-1.36)], the risk signatures could predict the prognosis of OS patients in most cases (Figure 5A). Then we constructed a regression model with the LASSO algorithm based on the above prognosis-related CFA markers, and the result showed the confidence interval under each lambda (Figure 5B). Then we modified the parameters of the LASSO regression model by cross-validation (Figure 5C). Based on the results of the above prognostic predictors, we selected three gene signatures CGREF1, CORT and RHBDL2 as key prognostic molecules and calculated a risk score for each sample based on the weight threshold of each signature, the risk score formula in LASSO-Cox regression analysis is: Risk-score = CGREF1*0.004 + CORT*0.004 + RHBDL2*0.002. In addition, we analyzed risk scores and prognostic assessments using multifactorial COX survival analysis using the TCGA database and GEO database respectively and plotted forest plots (Supplementary Figure S1). The analysis showed that CGREF1 in both GEO database and TCGA database and RHBDL2 in TCGA database showed promising predictions. Then we classified patients into high and low risk score groups using the median as the threshold (Figure 5D). Furthermore, the accuracy of risk score as a prognostic factor for OS was validated through ROC curve analysis using TCGA training set sample, the results of the survival analysis showed that there were significant differences between patients in groups with high and low scores (Figure 5E), Finally, the results were validated on a validation set cohort obtained by combining GSE21257 and GSE39058, which showed a significant difference in prognostic survival between the high and low risk score groups (Figures 5F–I). We then validated of gene expression in tissue of osteosarcoma patients. We demonstrated the results by IHC experiments, and observed that CGREF1, CORT and RHBDL2 proteins expression were high in the tissue of osteosarcoma patients, while the proteins were lowly expressed in normal tissue (Supplementary Figure S2).

### 3.5 CAFs signature could be used as an independent prognostic factor to predict tumor prognosis

**FIGURE 6:**
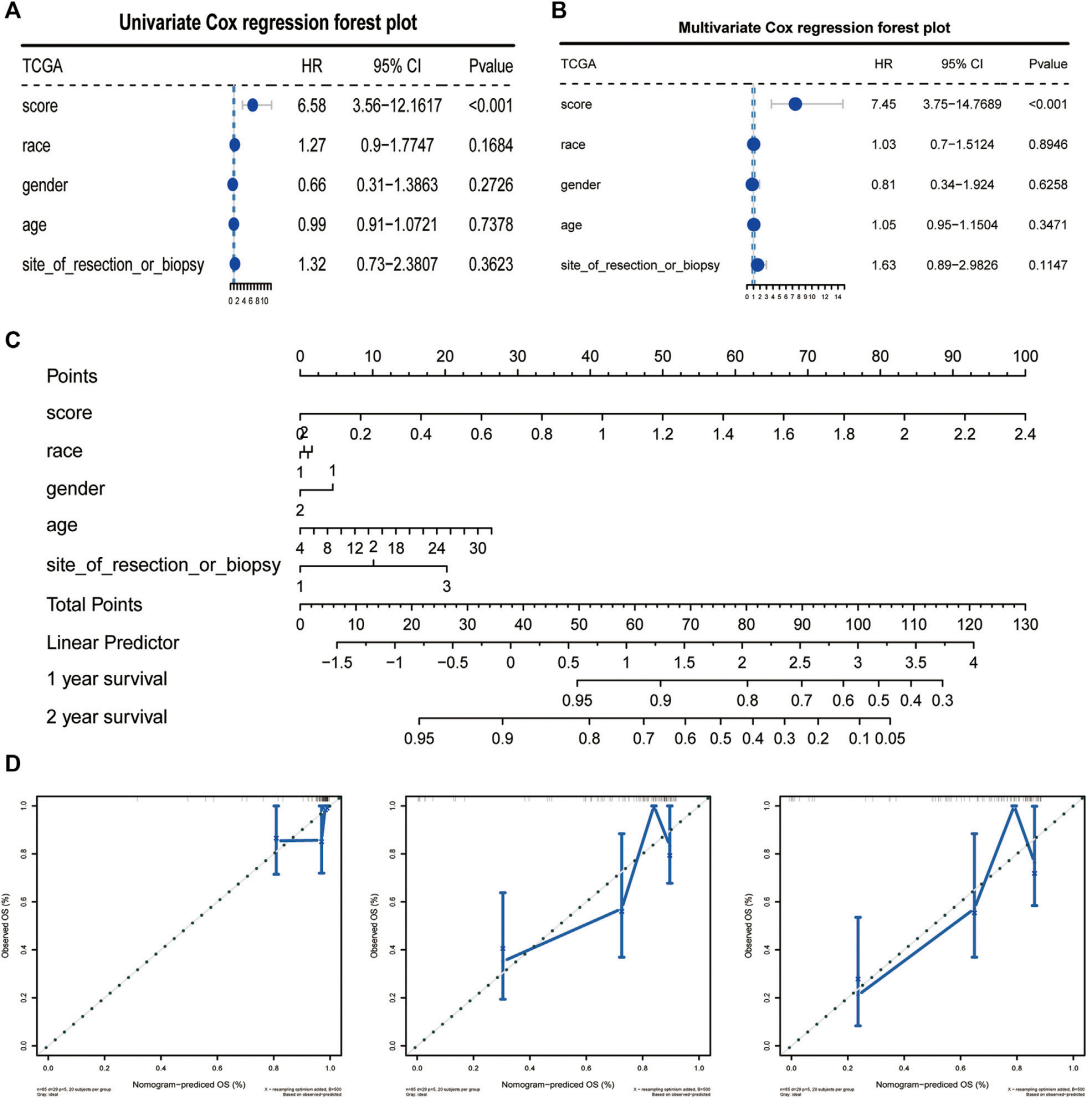
CAFs prognostic signatures could be used as an independent prognostic factor for OS. **(A, B)** CAFs risk score could be an independent prognostic factor confirmed by univariate and multivariate COX retrospective analyses. **(C)** Norton plot with clinical information. **(D)** Correction curves for one, three and 5 years.

We analyzed the correlation between CAF risk score and clinical characteristics, The results showed that as the risk score increased, the incidence of tumor metastasis increased correspondly, and there was a strong positive correlation between them, whereas the risk score was less correlated with other clinical differential characteristics, we then performed single-factor COX and multi-factor COX regression analyses based on the TCGA data set and the patients’ clinical characteristics to verify whether the CAF risk score could be used as an independent predictor of prognosis (Figures 6A, B). In addition, the Norton chart results showed that the CAF risk score had the greatest weighting and stronger prognostic power (Figures 6C, D). Above results showed that the CAF score could be used as an independent predictor of prognosis.

### 3.6 CAFs prognostic signature correlates with immune infiltration and tumor development

**FIGURE 7:**
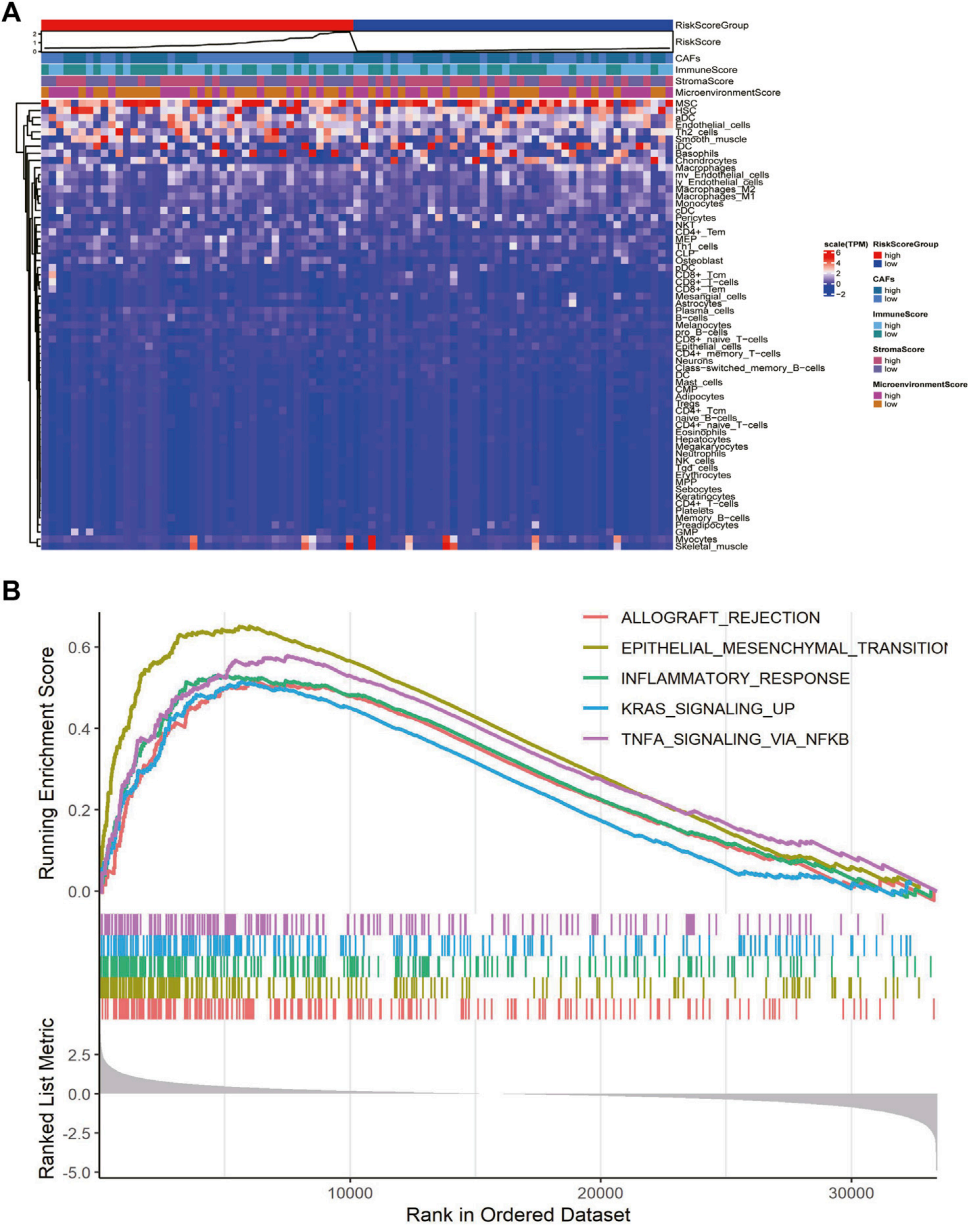
CAFs prognostic signatures associated with immune infiltration and tumor progression. **(A)** Heatmaps of immune cells and immune scores corresponding to high and low risk scores of CAFs. **(B)** Results of hallmark pathway enrichment between high and low score groups.

We explored the relationship between CAF risk score groupings and each tumor microenvironment cell (immune cells and stromal cells) and each immune score, and characterized its expression with a heat map, which showed that CAF infiltration did not correlate exactly positively with risk score, but patients with high-risk score had relatively low CAF infiltration (Figure 7A). Finally, based on the gene expression data profile of the high and low risk score groups, the R package clusterprofiler was used to enrich for 50 tumor-associated hallmark pathways, and we observed significant changes in the regulation of genes in the tumor-associated pathways (Figure 7B). In addition, we also analyzed the correlational relationship of the CAFs scores and the immune cells infiltration biomarker and immune suppression biomarkers using TCGA database (Figure 3S), and found a significant negative correlation between CAFs and CD 8, CD 25, and CD 206.

### 3.7 CAFs prognosis signature-associated drug resistance assessment

**FIGURE 8:**
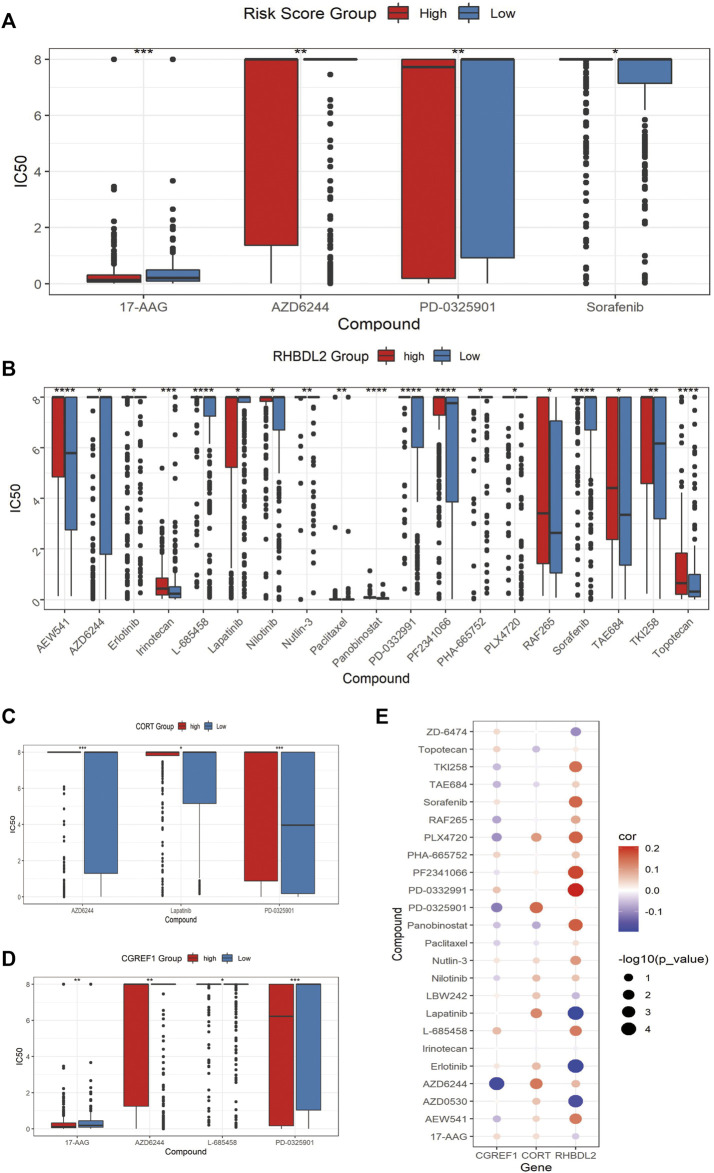
Assessment of associated resistance for CAFs prognostic signatures. **(A)** Drugs with significantly differentially in IC50 expression between high-risk and low-risk groups. **(B–D)**: Drugs with differential IC50 between high and low expression groups of RHBDL2, CORT, and CGREF1. **(E)** Correlation between RHBDL2, CORT, and CGREF1 expression and drug sensitivity.

Finally, we assessed the relationship between risk score and drug resistance in cell lines. From the CCLE (https://sites.broadinstitute.org/ccle) we downloaded information on 471 cell lines with expression profile data and resistance information (IC50) to 24 drugs, showing significant differences between the high and low scoring groups for 17-AAG, AZD6244, PD-0325901 and Sorafenib, there were significant differences between the high and low risk score groups (Figure 8A). We also analyzed the relationship between the expression of risk score model genes (RHBDL2, CORT, CGREF1) and drug resistance; we screened for significant differences in IC50 drug response between the high and low scoring groups based on median expression (Figures 8B–D); Finally, we observed a correlation between the IC50 response and gene expression, which confirmed the above findings (Figure 8E). To further validate the drug sensitivity in osteosarcoma cells, we used CCK-8 to measure the cellular viability. We demonstrate that the cell viability of HOS and MG63 cell lines was marked suppressed after 17-AAG, AZD6244, PD-0325901 and Sorafenib induction (Supplementary Figure S2).

## 5 Discussion

In recent years, numerous studies have shown that CAFs could play a regulatory role in tumors such as pancreatic and colorectal cancers by affecting stromal-tumor cell interactions, immune feedback, angiogenesis, and extracellular matrix remodeling (Kobayashi et al, 2019). Previous studies have reported a regulatory role of CAFs in the progression of osteosarcoma (Wang et al, 2019b; Zhao et al, 2021); however, studies on the overall function of CAFs in osteosarcoma are still limiting.

In the current study, we examined the role of CAF infiltration and clustering in predicting osteosarcoma prognosis. Based on XCELL calculation of enrichment scores and grouping by median scores, interestingly, we show for the first time that CAF high infiltration group has a better prognosis, and this difference may come from the dual pro-tumorigenic and tumor-suppressive roles of CAFs exhibited simultaneously in different tumor microenvironments (Wang et al, 2021). Our results also showed that the enrichment score of CAFs was positively and significantly correlated with stromal cell score and chondrocyte content, implying that CAFs might occupy a dominant position in the stromal component of osteosarcoma. Although studies surrounding the role of CAFs in tumors have gradually increased in recent years, there are few studies on the role of CAFs in the prognostic evaluation of osteosarcoma. In the present study, we evaluated that the high infiltration of CAFs plays a role in the prognosis of osteosarcoma, and the results suggest that CAFs risk score is an independent factor influencing the progression of osteosarcoma.

We identified numerous transcriptomic alterations and enrichment pathways in the CAFs highly infiltrated group compared with the low group. Enrichment by GO database and KEGG database showed that immune-related pathways such as B cell mediated immunity, humoral immune response are potentially critical pathways. We construct a survival risk model using CORT molecules, which are closely related to immune response (McCormick et al, 2015), and obtained good tumor risk prognosis, highlighting the potential role of immune-related pathways in the progression of osteosarcoma. In addition, whether greater benefit could be obtained with intervention of specific immune pathway inhibitors deserves to be further investigated.

Increasing evidence suggests that CAFs infiltrating in tumors might break down immunosuppression in tumors and further enhance tumor response to immunotherapy. Several current therapeutic strategies targeting CAFs include direct depletion of CAFs by immunotherapy targeting cell surface markers; normalizing activated CAFs; and targeting CAFs secreted extracellular matrix proteins or their associated signals (Chen and Song, 2019; Liu et al, 2019). Nanodrugs selectively target CAFs has also been shown to enhance infiltration of cytotoxic T-cells thereby to suppress tumor proliferation (Zhen et al, 2017). Studies have shown that the synergistic effect of immune checkpoint molecules and targeted CAFs may enhance the immunotherapeutic response by modulating the immunosuppressive environment (Feig et al, 2013). Our study indicates that CAFs in the extracellular matrix may be involved in the induction and formation of immune microenvironment during osteosarcoma progression and induce the phenotypic transformation of the tumor. Therefore, a better understanding of the interaction between CAFs and antitumor immunity would be beneficial for the establishment of effective immunotherapy. The emerging approaches such as single-cell RNA sequencing (Moncada et al, 2020) and spatial transcriptome (Xu et al, 2021) could provide a more comprehensive understanding of the spatial and temporal dynamics of CAFs interactions with tumor and immune cells and their specific roles in the osteosarcoma stroma.

With the function of producing inflammatory ligands, grown factors and extracellular matrix which could facilitate the tumor growth, resistance to treatment and immune escape, CAFs are always considered as the factor that promote tumorigenesis (Kalluri, 2016). However, with the establishment of new co-culture model and the development of single-cell RNA-sequencing (scRNA-seq) techniques, the subpopulation of CAFs has been found (Öhlund et al, 2017; Elyada et al, 2019; Hu et al, 2022). Öhlund et al (2017) found two different subclasses of CAFs. One group is distributed around tumor cells and highly expresses α-smooth muscle actin (α-SMA), which can produce connective tissue-forming matrix and is named myofibroblast CAFs (myCAFs). The other group is located far from the tumor cells, which is low in α-SMA expression and can secrete inflammatory factors such as IL-6, and is called inflammatory CAFs (iCAFs). In addition, based on scRNA-seq analysis, the study of Elyada et al identified three different CAFs subsets in pancreatic ductal adenocarcinoma. In addition to myCAFs and iCAFs which are previously identified, antigen presenting CAFs (apCAFs) that can utilize cell-expressed MHC II complexes and CD74 for antigen presentation have also been identified, which can present antigen to T-cells and play an anti-tumor role (Elyada et al, 2019). In this study, we found a prediction role of CAFs in the prognosis of OS, a further investigation might be need to clarify the prediction function of each subpopulation of CAFs in OS prognosis with the application of scRNA-seq analysis.

There are limitations present in the present study. First, we did not perform experiments to validate the relationships between CAFs and immune cells which were estimated by purely bioinformatic methods. Whether the conclusions obtained can be consistent with the real world should be viewed with caution. In the study, to reduce the possible bias brought by the analytical approach to CAFs, we scored CAFs using ordered categorical variables, resulting in a more consistent high CAF group or low CAF group. In this study, the data were processed by the XCELL computational algorithm after screening the MCPCOUNTER, XCELL, and EPIC algorithms, and the type abundance of cells was quantified by database-based cell labeling or deconvolution of cell mixtures based on gene expression matrices, and the above-mentioned methods allowed comparison between samples with identical cell types. In addition, multiple datasets from TCGA and GEO were used in this study to make the results obtained more robust, so the conclusions drawn in this study can still provide implications for interpreting the clinical and biological significance of CAFs. Second, large tumor tissues are a mixture of tumor cells themselves, stromal cells and immune cells as a whole, and transcriptomic changes and pathway alterations in each composed of them still need more experimental studies in the future. Meanwhile, this paper’s description of the role of CAFs on the clinical characteristics, clinical prognosis and prediction of the immune microenvironment of patients did not consider the subtypes and the spatial-temporal heterogeneity of CAFs. As there is a lack of universal markers for CAFs (Mueller and Fusenig, 2004; Biffi and Tuveson, 2021), the panorama of CAFs is difficult to be observed. ScRNA-seq technology has provided new insights in recent years. The identification of CAF subtypes in osteosarcoma using scRNA-seq technology has been reported (Huang et al, 2022). It is expecting that the gaps in this research area would be filled soon.

## Data Availability

The original contributions presented in the study are included in the article/Supplementary Material, further inquiries can be directed to the corresponding authors.

## Supplementary material

SUPPLEMENTARY FIGURE S1 Forest plots for assessments of risk scores and prognostic effect using multifactorial COX survival analysis for genes in TCGA database **(A)** and GEO database **(B)**.

SUPPLEMENTARY FIGURE S2 Validation of candidate genes and drug sensitivity. **(A–C)**: Validation of expression level of CGREF1, CORT and RHBDL2 in osteosarcoma and para-cancer tissue by IHC analysis. **(D–E)**: Verification of the sensitivity for 17-AAG, AZD6244, PD-0325901 and Sorafenib in HOS and MG63 cell lines by testing the cellular viability with CCK-8 measurement.

SUPPLEMENTARY FIGURE S3 The correlational relationship of the CAFs scores and the markers of immune cells infiltration and immune suppression. **(A)**: Correlation analysis on the CAFs score and the expression level of immune cells infiltration biomarker CD 8; **(B–D)**: Correlation analysis on the CAFs score and the expression level of immune suppression biomarker CD 4, CD 25, and CD 206.

## References

[B1] Biffi G. Tuveson D. A. (2021). Diversity and biology of cancer-associated fibroblasts. Physiol. Rev. 101 (1), 147–176. 10.1152/physrev.00048.2019 32466724 PMC7864232

[B2] Chen X. Song E. (2019). Turning foes to friends: Targeting cancer-associated fibroblasts. Nat. Rev. Drug Discov. 18 (2), 99–115. 10.1038/s41573-018-0004-1 30470818

[B3] Elyada E. Bolisetty M. Laise P. Flynn W. F. Courtois E. T. Burkhart R. A. (2019). Cross-species single-cell analysis of pancreatic ductal adenocarcinoma reveals antigen-presenting cancer-associated fibroblasts. Cancer Discov. 9 (8), 1102–1123. 10.1158/2159-8290.CD-19-0094 31197017 PMC6727976

[B4] Feig C. Jones J. O. Kraman M. Wells R. J. Deonarine A. Chan D. S. (2013). Targeting CXCL12 from FAP-expressing carcinoma-associated fibroblasts synergizes with anti-PD-L1 immunotherapy in pancreatic cancer. Proc. Natl. Acad. Sci. U. S. A. 110 (50), 20212–20217. 10.1073/pnas.1320318110 24277834 PMC3864274

[B5] Hu B. Wu C. Mao H. Gu H. Dong H. Yan J. (2022). Subpopulations of cancer-associated fibroblasts link the prognosis and metabolic features of pancreatic ductal adenocarcinoma. Ann. Transl. Med. 10 (5), 262. 10.21037/atm-22-407 35402584 PMC8987890

[B6] Huang X. Wang L. Guo H. Zhang W. Shao Z. (2022). Single-cell transcriptomics reveals the regulative roles of cancer associated fibroblasts in tumor immune microenvironment of recurrent osteosarcoma. Theranostics 12 (13), 5877–5887. 10.7150/thno.73714 35966586 PMC9373820

[B7] Ishii G. Ochiai A. Neri S. (2016). Phenotypic and functional heterogeneity of cancer-associated fibroblast within the tumor microenvironment. Adv. Drug Deliv. Rev. 99 (Pt B), 186–196. 10.1016/j.addr.2015.07.007 26278673

[B8] Kalluri R. (2016). The biology and function of fibroblasts in cancer. Nat. Rev. Cancer 16 (9), 582–598. 10.1038/nrc.2016.73 27550820

[B9] Kalluri R. Zeisberg M. (2006). Fibroblasts in cancer. Nat. Rev. Cancer 6 (5), 392–401. 10.1038/nrc1877 16572188

[B10] Kansara M. Teng M. Smyth M. Thomas D. (2014). Translational biology of osteosarcoma. Nat. Rev. Cancer 14 (11), 722–735. 10.1038/nrc3838 25319867

[B11] Keil L. (2020). Bone tumors: Primary bone cancers. FP Essent. 493, 22–26. 32573183

[B12] Kobayashi H. Enomoto A. Woods S. L. Burt A. D. Takahashi M. Worthley D. L. (2019). Cancer-associated fibroblasts in gastrointestinal cancer. Nat. Rev. Gastroenterol. Hepatol. 16 (5), 282–295. 10.1038/s41575-019-0115-0 30778141

[B13] Liotta L. A. Kohn E. C. (2001). The microenvironment of the tumour-host interface. Nature 411 (6835), 375–379. 10.1038/35077241 11357145

[B14] Liu T. Han C. Wang S. Fang P. Ma Z. Xu L. (2019). Cancer-associated fibroblasts: An emerging target of anti-cancer immunotherapy. J. Hematol. Oncol. 12 (1), 86. 10.1186/s13045-019-0770-1 31462327 PMC6714445

[B15] McCormick G. L. Shea K. Langkilde T. (2015). How do duration, frequency, and intensity of exogenous CORT elevation affect immune outcomes of stress? Gen. Comp. Endocrinol. 222, 81–87. 10.1016/j.ygcen.2015.07.008 26209864

[B16] Moncada R. Barkley D. Wagner F. Chiodin M. Devlin J. C. Baron M. (2020). Integrating microarray-based spatial transcriptomics and single-cell RNA-seq reveals tissue architecture in pancreatic ductal adenocarcinomas. Nat. Biotechnol. 38 (3), 333–342. 10.1038/s41587-019-0392-8 31932730

[B17] Mueller M. M. Fusenig N. E. (2004). Friends or foes - bipolar effects of the tumour stroma in cancer. Nat. Rev. Cancer 4 (11), 839–849. 10.1038/nrc1477 15516957

[B18] Öhlund D. Handly-Santana A. Biffi G. Elyada E. Almeida A. S. Ponz-Sarvise M. (2017). Distinct populations of inflammatory fibroblasts and myofibroblasts in pancreatic cancer. J. Exp. Med. 214 (3), 579–596. 10.1084/jem.20162024 28232471 PMC5339682

[B19] Robison L. Hudson M. (2014). Survivors of childhood and adolescent cancer: Life-long risks and responsibilities. Nat. Rev. Cancer 14 (1), 61–70. 10.1038/nrc3634 24304873 PMC6425479

[B20] Turley S. J. Cremasco V. Astarita J. L. (2015). Immunological hallmarks of stromal cells in the tumour microenvironment. Nat. Rev. Immunol. 15 (11), 669–682. 10.1038/nri3902 26471778

[B21] Wang J. W. Wu X. F. Gu X. J. Jiang X. H. (2019b). Exosomal miR-1228 from cancer-associated fibroblasts promotes cell migration and invasion of osteosarcoma by directly targeting SCAI. Oncol. Res. 27 (9), 979–986. 10.3727/096504018X15336368805108 30180920 PMC7848259

[B22] Wang S. He Z. Wang X. Li H. Liu X. S. (2019a). Antigen presentation and tumor immunogenicity in cancer immunotherapy response prediction. Elife 8, e49020. 10.7554/eLife.49020 31767055 PMC6879305

[B23] Wang Z. Yang Q. Tan Y. Tang Y. Ye J. Yuan B. (2021). Cancer-associated fibroblasts suppress cancer development: The other side of the coin. Front. Cell Dev. Biol. 9, 613534. 10.3389/fcell.2021.613534 33614646 PMC7890026

[B24] Xu R. Zhou X. Wang S. Trinkle C. (2021). Tumor organoid models in precision medicine and investigating cancer-stromal interactions. Pharmacol. Ther. 218, 107668. 10.1016/j.pharmthera.2020.107668 32853629 PMC7855432

[B25] Yahiro K. Matsumoto Y. (2021). Immunotherapy for osteosarcoma. Hum. Vaccin Immunother. 17 (5), 1294–1295. 10.1080/21645515.2020.1824499 33356848 PMC8078647

[B26] Zhao A. Zhao Z. Liu W. Cui X. Wang N. Wang Y. (2021). Carcinoma-associated fibroblasts promote the proliferation and metastasis of osteosarcoma by transferring exosomal LncRNA SNHG17. Am. J. Transl. Res. 13 (9), 10094–10111. 34650683 PMC8507050

[B27] Zhen Z. Tang W. Wang M. Zhou S. Wang H. Wu Z. (2017). Protein nanocage mediated fibroblast-activation protein targeted photoimmunotherapy to enhance cytotoxic T cell infiltration and tumor control. Nano Lett. 17 (2), 862–869. 10.1021/acs.nanolett.6b04150 28027646

[B28] Ziani L. Chouaib S. Thiery J. (2018). Alteration of the antitumor immune response by cancer-associated fibroblasts. Front. Immunol. 9, 414. 10.3389/fimmu.2018.00414 29545811 PMC5837994

