# Supplementary figures and images for: Cancer-associated fibroblast infiltration in osteosarcoma: the discrepancy in subtypes pathways and immunosuppression
Source: Front Pharmacol. 2023 Jun 27;14:1136960. doi: 10.3389/fphar.2023.1136960 (PMC10333483; doi:10.3389/fphar.2023.1136960)

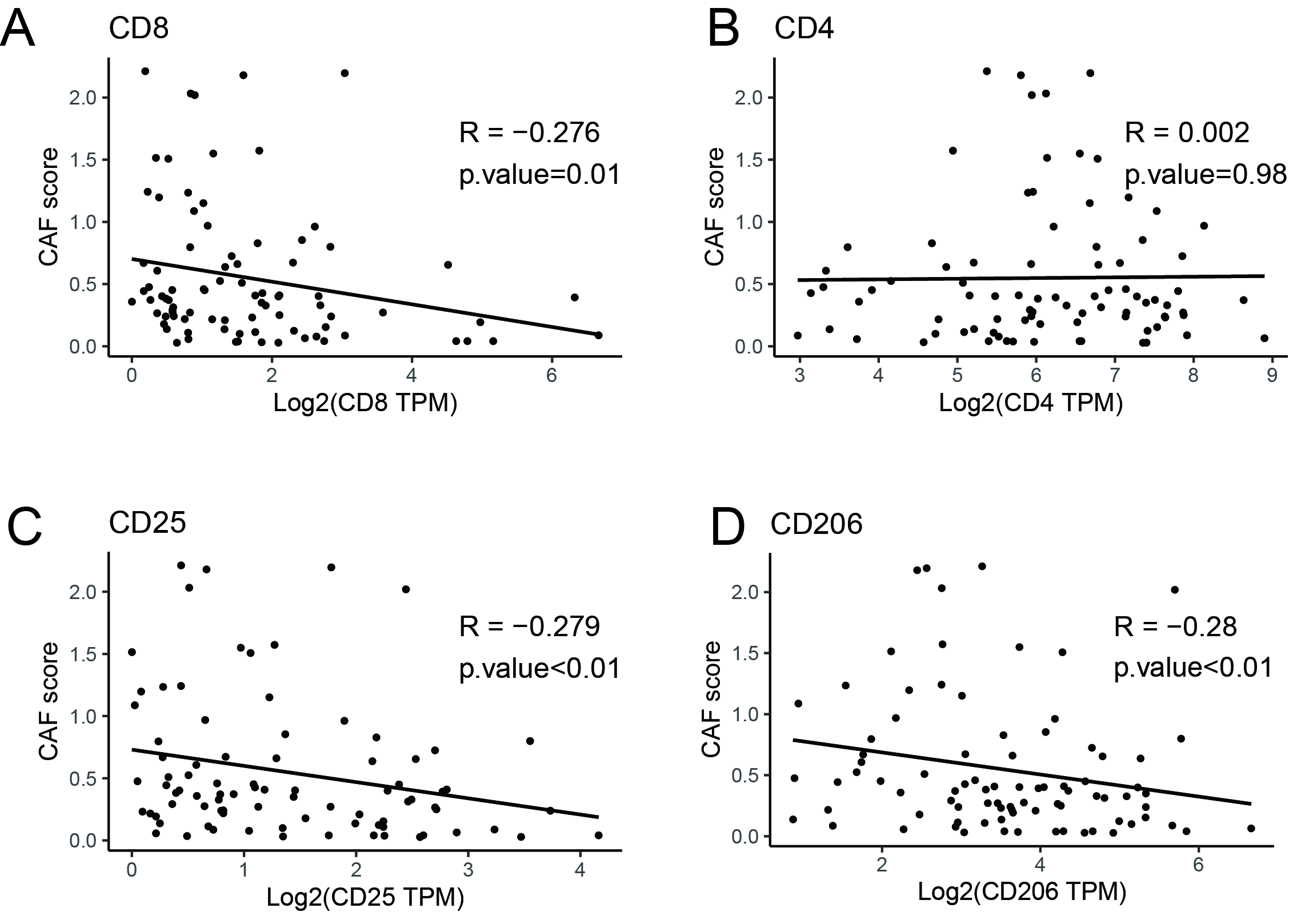

Supplement: Supplementary file 1 [file Image3.TIF]

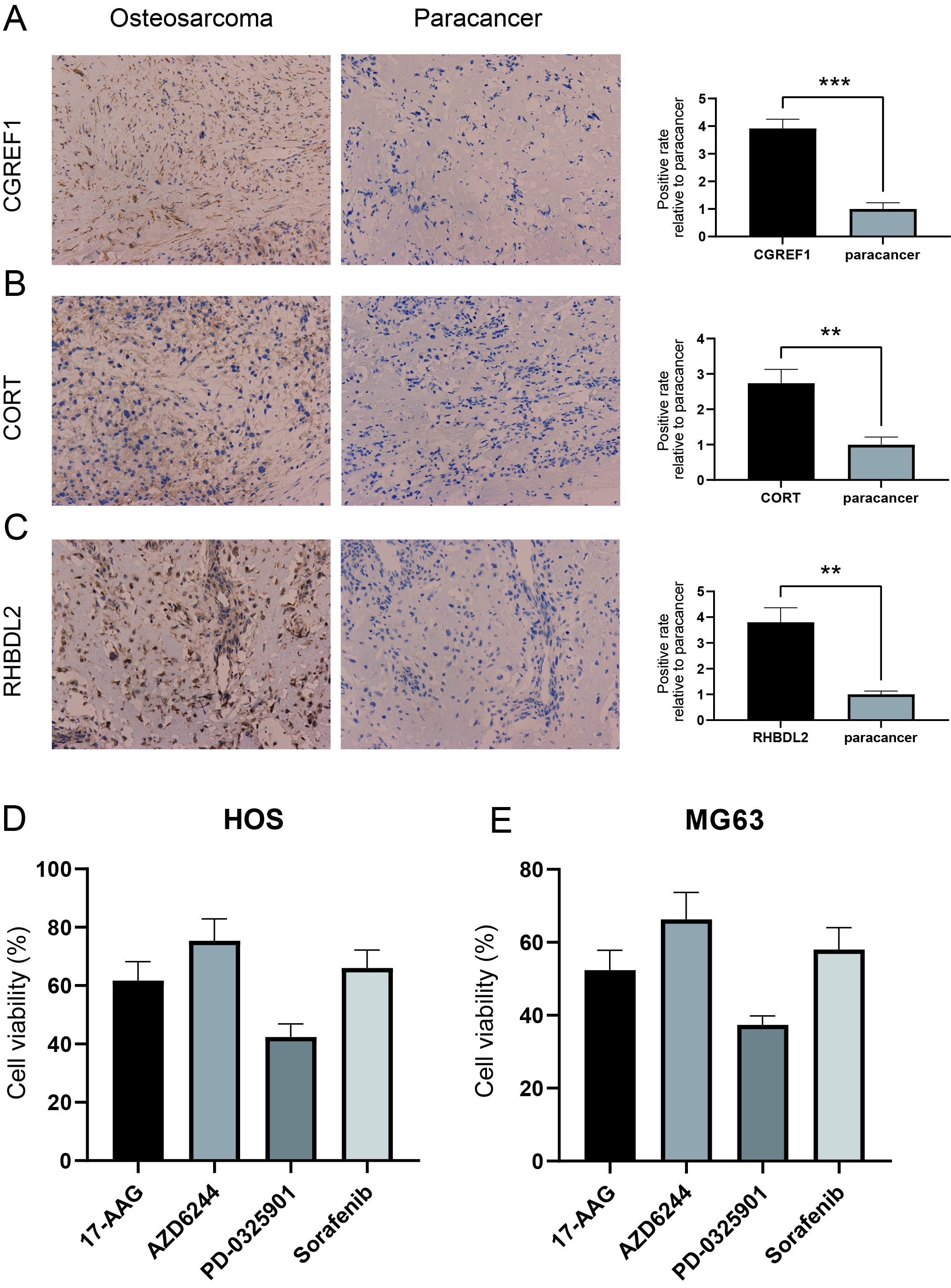

Supplement: Supplementary file 2 [file Image2.TIF]

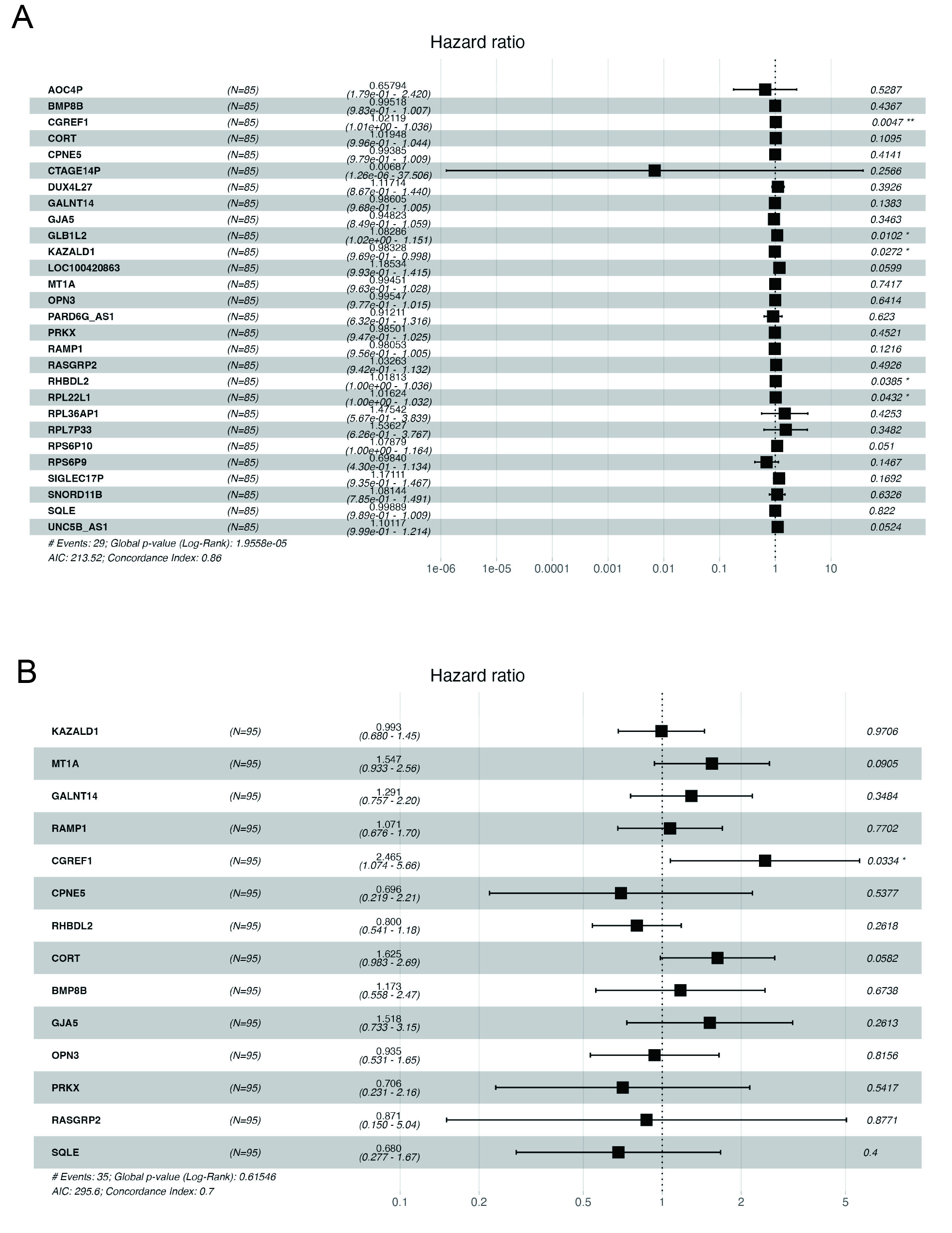

Supplement: Supplementary file 3 [file Image1.TIF]
